# Protein Intake Is Associated with Blood Pressure and Cholesterol Levels in Italian Older Adults: A Cross-Sectional Study

**DOI:** 10.3390/metabo13030431

**Published:** 2023-03-16

**Authors:** Hélio José Coelho-Júnior, Riccardo Calvani, Anna Picca, Matteo Tosato, Giulia Savera, Francesco Landi, Emanuele Marzetti

**Affiliations:** 1 Department of Geriatrics and Orthopedics, Università Cattolica del Sacro Cuore, 00168 Rome, Italy; coelhojunior@hotmail.com.br (H.J.C.-J.); giuliasavera1@gmail.com (G.S.); emanuele.marzetti@policlinicogemelli.it (E.M.); 2 Fondazione Policlinico Universitario “A. Gemelli”, IRCCS, 00168 Rome, Italy; picca@lum.it (A.P.); matteo.tosato@policlinicogemelli.it (M.T.); 3 Department of Medicine and Surgery, LUM University, 70100 Casamassima, Italy

**Keywords:** nutrition, diet, cardiovascular risk, cardiovascular disease, glucose, elderly

## Abstract

The present study was conducted to test the association between protein intake and blood pressure, glucose levels, and blood cholesterol in a large sample of Italian older adults. Longevity Check-up 7+ (Lookup 7+) is an ongoing project that started in June 2015. The project is conducted in unconventional settings (e.g., exhibitions, malls, health promotion campaigns) across Italy with the aim of fostering adoption of healthy lifestyles in the general population. For the present study, participants were eligible if they were 65+ years and provided written informed consent. Systolic (SBP) and diastolic blood pressure (DBP), and blood glucose and cholesterol levels were assessed. Protein intake was estimated using a 12-item food frequency questionnaire. Three-thousand four-hundred and four older adults were included in the study. The results of the linear regression showed an inverse association between protein intake (as a continuous variable) and DBP, and a positive correlation with blood cholesterol levels. The findings of the present study indicate that a high intake of protein was negatively associated with DBP and positively associated with total blood cholesterol levels in a large cohort of Italian older adults, after adjustment for numerous covariates.

## 1. Introduction

Cardiovascular disease (CVD) is the leading cause of morbidity and mortality globally, with a yearly death toll of almost 20 million [1]. The incidence of CVD increases with age, such that more than 70% of those aged 60+ years are affected [2,3]. The burden of CVD in older adults is especially concerning given the association of CVD with hospitalization, healthcare costs [2,3], and mortality [2,3]. The high prevalence of CVD in old age results from both cardiovascular aging [4] and lifetime exposure to risk factors, such as high blood pressure (BP), abnormal glucose metabolism, hyperlipidemia, environmental pollution, and unhealthy behaviors [5]. 

Changes in lifestyle habits are a cornerstone in the prevention and management of cardiometabolic diseases [6,7,8]. Studies have found that specific dietary patterns (e.g., Mediterranean and DASH diets) may positively affect cardiovascular and metabolic parameters [9,10]. However, differences in food availability and quality, as well as cooking methods, limit the widespread implementation of these nutritional regimens [11]. Individual macronutrients also have an impact on cardiovascular health. Besides lipids and carbohydrates, a high intake of dietary protein may increase the risk of CVD [12]. However, some amino acids (AAs) have hypotensive effects. For instance, l-arginine serves as a substratum for nitric oxide production, which acts as a potent vasodilator [13]. Moreover, an increased consumption of tyrosine is negatively associated with BP [14].

Studies on the relationship between high protein intake and cardiometabolic parameters have produced mixed results [15,16,17,18,19,20,21,22,23,24,25,26]. Most of these findings were obtained in small samples of adults from different age groups, while studies exclusively based on the old population are scarce. Furthermore, high protein intake has been commonly defined as a protein ingestion greater than the current recommended dietary allowance (RDA, ≥0.8 g of protein per kg of body weight (BW) per day) [27,28]. However, these recommendations have been questioned, given that the RDA is based on nitrogen balance studies and no specific recommendations for older adults are available [27,28]. For this reason, several investigations studies have estimated protein intake levels based on the dietary habits of the studied population [26], using tertiles [14,19,23] or quintiles [21].

To expand the knowledge on the subject, the present study was conducted to test the association between protein intake and cardiometabolic risk factors, including BP, glucose levels, and blood cholesterol, in a relatively large sample of Italian older adults. We also explored the relationship between cardiometabolic risk factors and high protein ingestion estimated according to tertiles and quintiles to identify values of protein intake associated with cardiometabolic health.

## 2. Experimental Design

Data of the present investigation were gathered from the Longevity Check-up 7+ (Lookup 7+) project database. Sampling characteristics, procedures, and other results have been published elsewhere [29,30,31,32,33,34]. Lookup 7+ is an ongoing initiative developed by the Department of Geriatrics of the Fondazione Policlinico “Agostino Gemelli” IRCCS at the Università Cattolica del Sacro Cuore (Rome, Italy). The project was designed to foster healthy and active aging by raising awareness among the general public on the importance of modifiable risk factors for chronic diseases [29,30,31,32,33,34].

Recruitment was conducted among people visiting public spaces (e.g., exhibitions, shopping centers) and those adhering to prevention campaigns promoted by our institution. As previously described, recruitment activities were carried out in small (<100,000 inhabitants), medium (100,000–250,000 inhabitants), and large cities (>250,000 inhabitants) to achieve a comprehensive geographic coverage of mainland Italy and major islands [29]. In large cities, participants were recruited in different locations to maximize the representation of the sociodemographic characteristics of inhabitants. The Lookup 7+ protocol was approved by the Ethics Committee of the Università Cattolica del Sacro Cuore (protocol #: A.1220/CE/2011) and each participant provided written informed consent prior to enrolment. The manuscript was prepared in compliance with the STrengthening the Reporting of Observational studies in Epidemiology (STROBE) guidelines for observational studies [35].

## 3. Procedure

### 3.1. Participants

From 1 June 2015 to 31 October 2021, 13,515 community-dwelling adults aged 18+ years participated in the study. Exclusion criteria were inability or unwillingness to provide written informed consent, self-reported pregnancy, and inability to perform the physical function tests as per the study protocol. For the present investigation, only people aged 65+ years, with body mass index (BMI) values ≥18.5 kg/m^2^ and no missing data for the study variables, were included, totaling 3404 participants (10,111 excluded).

Each participant received a structured interview to collect information on lifestyle habits, followed by measurement of anthropometric parameters, including height and weight. The BMI was calculated as the ratio between body weight (kg) and the square of height (m^2^). An oscillometric monitor was used to measure BP (Omron M6 electronic sphygmomanometer, Omron, Kyoto, Japan) [36]. Glucose levels and total blood cholesterol were measured from capillary blood samples using disposable electrode strips based on a reflectometric system with a portable device (MultiCare-In, Biomedical Systems International Srl, Florence, Italy) [37]. Participants were asked if they were fasting for at least 8 h. A food frequency questionnaire (FFQ) adapted from Landi et al. [37] was used to collect information on how often in a week participants consumed a standardized portion size of a list of 12 foods, including meat, meat derivatives, fish, eggs, milk, cheese, yogurt, pasta, bread, rice, vegetables, and cereals. Portion size was estimated based on the Italian standard portion reference [37]. The mean daily intake of protein was calculated by multiplying the consumption frequency of a food item by the protein content of its standard portion [37], then dividing by seven (the days of a week), followed by the sum of all applicable items. Smoking status was defined as follows: current smoker (has smoked 100+ cigarettes in lifetime and currently smokes cigarettes), and no current smoker. Regular participation in physical activity was considered as involvement in leisure-time physical activity at least twice a week, 30 min per session, during the past year [37]. Accordingly, participants were considered either physically active or inactive. Participants provided information on the use of antihypertensive, cholesterol-lowering, and antidiabetic drugs.

### 3.2. Statistical Analysis

The normal distribution of variables was ascertained via the Shapiro–Wilk test. Continuous variables are expressed as the mean ± standard deviation (SD) or absolute numbers, percentages. Tertiles and quintiles for BW-adjusted daily protein intake were: ≤0.79, 0.80–0.99, and ≥1.00; and ≤0.42, 0.43–0.52, 0.53–0.60, 0.61–0.70, and ≥0.71. Regression analysis was conducted to test the association between protein intake and cardiometabolic risk factors. Scatterplots were constructed using the variables significantly associated. Pearson’s correlation was conducted to quantify the association among the variables analyzed in the Scatterplots. The final model was adjusted for age, sex, BMI, energy intake, sodium, potassium, calcium, magnesium, physical activity, active smoking, fasting state (blood glucose), and antihypertensive (for systolic (SBP) and diastolic BP (DBP)), cholesterol-lowering (for total blood cholesterol), and antidiabetic (for blood glucose) drugs. 

Significance was set at 5% (*p* < 0.05) for all tests. All analyses were performed using the SPSS software (version 23.0, SPSS Inc., Chicago, IL, USA).

## 4. Results

Three-thousand four-hundred and four older adults were analyzed in the present study. The main characteristics of participants according to quintiles and tertiles of protein intake are shown in Table 1 and Table 2, respectively. SBP (range 90−200 mmHg), DBP (50−70 mmHg), and BMI (18.5−32.0 kg/m^2^) were within normal ranges. The studied characteristics did not differ depending on the method used to categorize protein intake. Those in the highest tertile (≥1.0 g/kg BW/day) and quintiles (0.61−0.70 and ≥0.71 g/kg BW/day) of protein intake were older than participants with lower consumption. Energy and micronutrient intake parameters increased across tertiles and quintiles of protein consumption. In contrast, protein intake was inversely related to BMI values. 

### 4.1. Association of Protein Intake with Cardiovascular Risk Factors

Specific patterns of associations were observed between protein intake and cardiometabolic parameters. When participants were stratified according to quintiles of protein intake, those who consumed ≥0.71 g/kg BW/day had lower DBP values and higher blood cholesterol levels in comparison with all other groups. When participants were stratified in tertiles, those with a protein consumption of 0.80−0.99 g/kg BW/day showed higher blood cholesterol levels than participants with an intake ≤0.79 g/kg BW/day. Participants who consumed ≥1.00 g/kg BW/day had higher blood cholesterol levels when compared with the other two groups. DBP values were lower in participants with a protein intake of 0.80−0.99 g/kg BW/day than in those in the lowest tertile. No differences in DBP were observed among participants with a protein consumption ≥1.00 g/kg BW/day and the other protein ingestion groups. Sex distribution and the use of antihypertensive medications differed across tertiles and quintiles of protein intake, whereas smoking and physical activity habits differed according to quintiles.

### 4.2. Linear Regression

**Figure 1:**
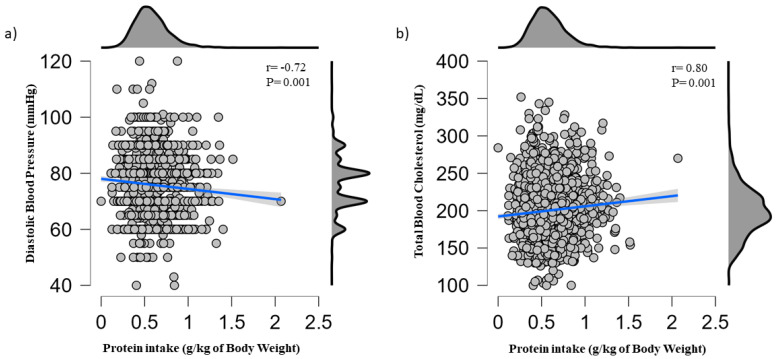
Scatter plot of the association between daily protein intake and (**a**) diastolic blood pressure and (**b**) total blood cholesterol.

Results of the linear regression analysis for the association between protein intake and BP, total blood cholesterol, and glucose levels are shown in Table 3, Table 4 and Table 5, respectively. Protein intake classified according to either quintiles or tertiles was not significantly associated with SBP, DBP, blood cholesterol, or glucose levels after adjusting for covariates. However, an inverse association with DBP (β = −4.925; 95% confidence interval (CI) −9.455, −0.394) and a positive correlation with blood cholesterol levels (β = 17.139; 95% CI 2.021, 32.256) were found when protein intake was analyzed as a continuous variable. Figure 1 shows scatterplots for the associations between protein intake and DBP (Figure 1a) and total blood cholesterol (Figure 1b). 

## 5. Discussion

The findings of the present study indicate that a high intake of protein is negatively associated with DBP and positively correlated with blood cholesterol levels in a large and relatively unselected sample of Italian older adults. These associations were significant when protein intake was analyzed as a continuous variable, but not when quintiles or tertiles of protein of intake were used.

Some studies that have investigated the relationship between protein consumption and BP support our results. Mente et al. [25] examined more than 100,000 individuals from 18 countries and found that a high protein intake was negatively associated with BP. Similar findings were reported by other investigations that also assessed large cohorts, such as the INTERSALT [15] and Framingham [16,19] studies. Umesawa et al. [38] observed similar patterns of associations in an Asian population.

However, other studies found no significant associations between protein intake and BP-related parameters. For instance, absolute protein intake was found to be unrelated to age-related changes in BP and hypertension incidence in Dutch adults [21,22]. Similar findings were reported by Liu et al. [24] regarding the prevalence of hypertension in poorly nourished rural Chinese people. Increased protein intake also failed to ameliorate hemodynamic parameters in randomized clinical trials. Indeed, Hodgson et al. [39] did not find differences in BP values between older adults on protein supplementation or isocaloric supplement for two years.

Differences across studies might be attributed to sample characteristics (e.g., age, sex distribution) [15], amount and quality of dietary protein [23,38,40], hypertension status [21], and the covariables used to adjust the analyses [19,21,23,40]. Interestingly, stronger associations between BP and protein consumption have been observed in older women [15] with untreated hypertension [21]. In contrast, the present study examined a cohort of young older adults [8], prevalently composed of men with BMI and BP levels within normal ranges. 

An interesting scenario was recently offered by He et al. [40], who found a U-shaped relationship between protein consumption and hypertension, with the lowest prevalence of hypertension in individuals with the highest protein intake. Mehrabani et al. [41] proposed an alternative model based on an inverse dose–response association between protein consumption and BP levels. These data might explain why DBP, but not SBP, and continuous, but not categorical data, were significantly associated with protein intake.

Another important result of the present study was that protein intake and blood cholesterol levels were positively correlated. These findings are not supported by most prior research, given that investigations observed negative [17,26] or null [18,42] associations. However, Mente et al. [25] noted that a high intake of protein was associated with elevated blood cholesterol levels [25]. Lifestyle interventions based on protein-rich diets have also been investigated as a non-pharmacological lipid-lowering strategy with promising results [26,43]. 

There are some possible explanations for our observations. Increasing protein intake raises the concern for potential harm to blood lipid profile [44] and CVD risk [12] due to a possible simultaneous increase in fat intake. For this reason, intervention studies testing high-protein diets typically involve low energy from saturated fats (<10%) [43]. Second, protein sources are an important parameter to take into consideration [44]. Red meat is a common source of dietary protein [45,46] and evidence has shown that the prevalence of hyperlipidemia increases according to meat consumption [47]. Unprocessed and processed red meat increases the risk of type II diabetes [45] and death [46], while diets containing low-fat sources of protein (e.g., nuts, dairy products) are associated with a significantly lower risk of adverse events [45,46]. In contrast, high fish consumption is associated with a low monocyte/high-density lipoprotein cholesterol ratio, thereby reducing CVD risk [48].

An additional explanation for our data involves the AA composition of dietary protein. Several AAs, chiefly methionine and cysteine, might influence blood cholesterol levels [49,50,51]. Cysteine is the major precursor of taurine [52,53]. Fish and seafood (e.g., oysters) are the main sources of taurine, with small concentrations found in poultry (e.g., chicken and turkey) [52,53]. Taurine supplementation reduces total and low-density lipoprotein (LDL)-cholesterol in rats [54] and humans [52]. Such a hypocholesterolemic effect seems to occur through the activation of 7-hydroxylase, which accelerates the catabolism of cholesterol into bile acid [53]. 

Leucine might have hypocholesterolemic effects. Leucine supplementation for 14 weeks decreased total and LDL–cholesterol levels in mice fed a high-fat diet [55]. In rats, leucine reduced systemic LDL–cholesterol levels [56]. Furthermore, leucine attenuated aging-related impairments in endothelium-dependent and independent vasodilation and collagen deposition in the medial layer of the aorta, and abolished inflammatory cell infiltration in male mice [57]. Decreased systemic inflammation was also described in rats supplemented with leucine [56].

The findings of the present study highlight the importance of professional nutritional counseling to ensure that protein intake is not accompanied by an elevated consumption of dietary components, such as fat, that might raise cholesterol levels and, thereby, cardiovascular risk. A possible strategy to possibly avoid the harmful effects of an increased consumption of other macronutrients might be utilize high-protein, low-fat foods (e.g., lean red meat) [58,59,60]. Despite our efforts of using different methods to identify high protein intake levels that were more associated with cardiometabolic factors, we were unable to establish cutoff values in our population. Such a scenario suggests that an accurate and close monitoring of nutrient intake is necessary to minimize the unwanted effects of diet on cardiometabolic risk. Therefore, the results of our study should be complemented with those of randomized clinical trials specifically designed to test the effects of various levels of protein intake on cardiometabolic parameters in older adults. 

A major limitation of the present study is that the FFQ used only included weekly consumption of 12 food groups. It is therefore possible that participants either ate foods that were not included in the FFQ or under-reported their dietary intake, which impacted the accuracy of protein intake estimation. Second, participants were relatively young (65–74 years) [61] community-dwelling older Caucasians, and extrapolation to people in other age groups (e.g., old-old adults ≥75 years) or ethnicities should be made with caution. Third, participants were evaluated while they were attending an event. Thus, the possibility the evaluation setting could have influenced the assessment results cannot be excluded. Fourth, a high-protein diet was found to reduce HbA1C, but not blood glucose levels [44]. However, HbA1C was not measured in our study. Fifth, specific patterns of associations have been observed between protein sources and cardiometabolic parameters [62,63]. This aspect was not investigated in the present study. Sixth, participants with cardiovascular or cerebrovascular disease were not excluded and the possibility that this might have influenced our results cannot be ruled out. Finally, the cross-sectional design of the study does not allow any inference to be drawn on the time course of changes in the analyzed variables or on cause-effect relationships.

In conclusion, the findings of the present study indicate that a high intake of protein was negatively associated with DBP and positively associated with total blood cholesterol levels in a relatively large cohort of older Italian adults after adjustment for numerous covariates.

## Figures and Tables

**Table 1:** Main characteristics of study participants according to quintiles of daily protein intake (*n* = 3404).

	Quintiles of Daily Protein Intake (g/kg of Body Weight)				
	≤0.42 (*n* = 681)	0.43−0.52 (*n* = 680)	0.53−0.60 (*n* = 682)	0.61−0.70 (*n* = 680)	≥0.71 (*n* = 681)
Age, years	72.1 ± 5.4	72.6 ± 5.8	72.7 ± 5.7	73.1 ± 5.6 ^a^	73.1 ± 5.7 ^a^
Weight, kg	79.3 ± 13.4	74.7 ± 12.6 ^a^	71.3 ± 10.9 ^ab^	67.9 ± 10.5 ^abc^	60.6 ± 9.8 ^abcd^
Height, m	1.66 ± 0.9	1.65 ± 0.9	1.63 ± 0.8 ^ab^	1.61 ± 0.9 ^abc^	1.58 ± 0.8 ^abcd^
BMI, kg/m^2^	28.6 ± 4.4	27.1 ± 4.1 ^a^	26.5 ± 3.6 ^ab^	25.9 ± 3.5 ^abc^	24.0 ± 3.2 ^abcd^
Sex, female	295, 43.3	290, 42.6	357, 52.3	405, 59.5	528, 77.5 *
Energy intake, kcal	388.1 ± 104.6	492.2 ± 95.2 ^a^	546.7 ± 98.3 ^ab^	612.2 ± 103.9 ^abc^	688.4 ± 124.8 ^abcd^
Protein, g	27.2 ± 6.3	35.0 ± 5.9 ^a^	39.7 ± 6.0 ^ab^	44.6 ± 7.0 ^abc^	51.9 ± 8.6 ^abcd^
Protein, g/kg	0.34 ± 0.06	0.47 ± 0.02 ^a^	0.56 ± 0.02 ^ab^	0.65 ± 0.02 ^abc^	0.85 ± 0.14 ^abcd^
Calcium, mg	178.3 ± 100.9	235.0 ± 107.6 ^a^	284.0 ± 116.2 ^ab^	331.7 ± 120.7 ^abc^	383.5 ± 126.4 ^abcd^
Magnesium, mg	32.6 ± 10.0	39.6 ± 10.0 ^a^	44.0 ± 9.8 ^ab^	48.3 ± 10.3 ^abc^	54.3 ± 11.2 ^abcd^
Sodium, mg	417.2 ± 195.4	559.1 ± 217.3 ^a^	619.7 ± 230.9 ^ab^	722.7 ± 251.5 ^abc^	825.9 ± 317.1 ^abcd^
Potassium, mg	356.9 ± 100.4	449.4 ± 102.6 ^a^	503.7 ± 104.3 ^ab^	555.9 ± 118.1 ^abc^	641.6 ± 144.3 ^abcd^
SBP, mmHg	130.9 ± 15.0	130.4 ± 14.9	131.7 ± 17.0	130.2 ± 16.0	130.0 ± 17.2
DBP, mmHg	76.4 ± 9.3	76.5 ± 9.8	76.3 ± 9.5	75.6 ± 9.4	74.6 ± 9.7 ^abc^
Total blood cholesterol, mg/dL	199.3 ± 34.3	196.8 ± 33.2	196.9 ± 31.4	201.3 ± 31.5	205.4 ± 33.8 ^abc^
Blood glucose, mg/dL	107.7 ± 27.0	107.7 ± 26.0	107.2 ± 25.5	105.5 ± 22.5	105.4 ± 24.8
Smoking, yes	105, 15.4	101, 14.8	84, 12.3	88, 12.4	71, 10.4 *
Physical activity, yes	135, 19.8	167, 24.5	182, 26.6	165, 24.2	164, 24.0 *
Antihypertensive drug(s), yes	405, 12.0	377, 11.2	373, 11.1	355, 10.5	310, 9.2 *
Cholesterol-lowering drug(s), yes	235, 34.5	230, 33.8	232, 34.0	218, 32.0	209, 30.6
Antidiabetic drug(s), yes	65, 9.5	63, 9.2	53, 7.7	58, 8.5	40, 5.8

Data are shown as mean ± standard deviation or absolute number, percentage. ^a^ *p* < 0.05 vs. *p* ≤ 0.42, ^b^ *p* < 0.05 vs. 0.43–0.52, ^c^ *p* < 0.05 vs. 0.53−0.60, ^d^ *p* < 0.05 vs. 0.61–0.70. * *p* < 0.05 according to chi-square tests. Abbreviations: BMI, body mass index, DBP, diastolic blood pressure, SPB, systolic blood pressure.

**Table 2:** Main characteristics of study participants according to tertiles of daily protein intake (*n* = 3404).

	Tertiles of Daily Protein Intake (g/kg of Body Weight)		
	≤0.79 (*n* = 2993)	0.80−0.99 (*n* = 304)	≥1.00 (*n* = 107)
Age, years	72.6 ± 5.6	72.9 ± 5.8	74.6 ± 6.5 ^ab^
Weight, kg	72.5 ± 12.6	59.9 ± 8.7 ^a^	52.5 ± 7.5 ^ab^
Height, m	1.64 ± 0.9	1.58 ± 0.7 ^a^	1.55 ± 0.8 ^ab^
BMI, kg/m^2^	26.8 ± 4.0	23.8 ± 3.1 ^a^	21.7 ± 2.8 ^ab^
Sex, female	1526, 50.9	237, 77.9	87, 81.3 *
Energy intake, kcal	521.0 ± 133.4	695.0 ± 118.9 ^a^	791.2 ± 117.1 ^ab^
Protein, g	37.6 ± 9.4	52.2 ± 7.9 ^a^	59.8 ± 8.7 ^ab^
Protein, g/kg	0.52 ± 0.13	0.87 ± 0.05 ^a^	1.14 ± 0.14 ^ab^
Calcium, mg	266.0 ± 128.3	381.5 ± 125.2 ^a^	457.9 ± 110.3 ^ab^
Magnesium, mg	42.0 ± 11.8	54.0 ± 10.8 ^a^	62.9 ± 9.8 ^ab^
Sodium, mg	595.3 ± 259.4	833.2 ± 306.5 ^a^	962.2 ± 363.8 ^ab^
Potassium, mg	477.4 ± 133.9	646.7 ± 139.6 ^a^	739.1 ± 146.9 ^ab^
SBP, mmHg	130.7 ± 15.8	129.2 ± 16.3	131.2 ± 18.4
DBP, mmHg	76.1 ± 9.5	74.1 ± 9.3 ^a^	74.6 ± 10.2
Total blood cholesterol, mg/dL	199.1 ± 32.7	205.7 ± 33.7 ^a^	209.6 ± 35.9 ^ab^
Blood glucose, mg/dL	106.9 ± 25.1	104.0 ± 21.3	108.9 ± 37.7
Smoking, yes	404, 13.4	35, 11.5	7, 6.5
Physical activity, yes	714, 23.8	65, 21.3	24, 22.4
Antihypertensive drug(s), yes	1631, 55.5	137, 45.0	32, 30.0 *
Cholesterol-lowering drug(s), yes	997, 33.3	85, 30.0	31, 29.0
Antidiabetic drug(s), yes	252, 8.4	16, 5.2	10, 9.3

Data are shown as mean ± standard deviation or absolute number, percentage. ^a^ *p* < 0.05 vs. *p* ≤ 0.79, ^b^ *p* < 0.05 vs. 0.80–0.99. * *p* < 0.05 according to chi-square tests. Abbreviations: BMI, body mass index, DBP, diastolic blood pressure, SPB, systolic blood pressure.

**Table 3:** Association between protein intake and blood pressure.

	Unadjusted β (95% CI)	Adjusted * β (95% CI)		Unadjusted β (95% CI)	Adjusted * β (95% CI)		Unadjusted β (95% CI)	Adjusted * β (95% CI)		Unadjusted β (95% CI)	Adjusted * β (95% CI)
SPB, mmHg											
≤0.42 g/kg of BW	1.0 (Reference)	1.0 (Reference)	≤0.79 g/kg of BW	1.0 (Reference)	1.0 (Reference)	0.80−0.99 g/kg of BW	1.0 (Reference)	1.0 (Reference)	Protein intake g/kg of BW	−1.546 (−4.431, 1.339)	−2.031 (−9.593, 5.531)
≥0.71 g/kg of BW	−0.227 (−0.667, 0.212)	−0.284 (−1.332, 0.764)	≥1.00 g/kg of BW	−0.427 (−1.705, 0.851)	1.031 (−1.488, 3.549)	≥1.00 g/kg of BW	−0.183 (−1.149, 0.782)	0.287 (−0.891, 1.464)			
DBP, mmHg											
≤0.42 g/kg of BW	1.0 (Reference)	1.0 (Reference)	≤0.79 g/kg of BW	1.0 (Reference)	1.0 (Reference)	0.80−0.99 g/kg of BW	1.0 (Reference)	1.0 (Reference)	Protein intake, g/kg of BW	−3.661 (−5.380, −1.943)	−4.925 (−9.455, −0.394)
≥0.71 g/kg of BW	−0.437 (−0.696, −0.178)	−0.236 (−0.864, 0.392)	≥1.00 g/kg of BW	−1.259 (−2.025, −0.494)	3.258 (−0.009, 6.525)	≥1.00 g/kg of BW	−0.838 (−1.417, −0.259)	−0.168 (−0.877, 0.542)			

* Models were adjusted for age, sex, body mass index, energy intake, sodium, potassium, calcium, magnesium, physical activity, active smoking, and antihypertensive therapy. Abbreviations: BMI, body mass index, BW, body weight, CI, confidence interval, DBP, diastolic blood pressure, SPB, systolic blood pressure.

**Table 4:** Association between protein intake and total blood cholesterol.

	Unadjusted β (95% CI)	Adjusted * β (95% CI)		Unadjusted β (95% CI)	Adjusted * β (95% CI)		Unadjusted β (95% CI)	Adjusted * β (95% CI)		Unadjusted β (95% CI)	Adjusted * β (95% CI)
Cholesterol, mg/dL											
≤0.42 g/kg of BW	1.0 (Reference)	1.0 (Reference)	≤0.79 g/kg of BW	1.0 (Reference)	1.0 (Reference)	0.80−0.99 g/kg of BW	1.0 (Reference)	1.0 (Reference)	Protein intake, g/kg of BW	14.085 (8.188, 19.983)	17.139 (2.021, 32.256)
≥0.71 g/kg of BW	1.521 (0.595, 2.446)	−0.881 (−3.061, 1.298)	≥1.00 g/kg of BW	5.818 (3.174, 8.462)	−0.418 (−1.381, 0.546)	≥1.00 g/kg of BW	4.122 (2.212, 6.218)	2.400 (−0.012, 4.812)			

* Models were adjusted for age, sex, body mass index, energy intake, physical activity, active smoking, cholesterol-lowering therapy. Abbreviations: BMI, body mass index, BW, body weight, CI, confidence interval.

**Table 5:** Association between protein intake and blood glucose.

	Unadjusted β (95% CI)	Adjusted * β (95% CI)		Unadjusted β (95% CI)	Adjusted * β (95% CI)		Unadjusted β (95% CI)	Adjusted * β (95% CI)		Unadjusted β (95% CI)	Adjusted * β (95% CI)
Glucose, mg/dL											
≤0.42 g/kg of BW	1.0 (Reference)	1.0 (Reference)	≤0.79 g/kg of BW	1.0 (Reference)	1.0 (Reference)	0.80−0.99 g/kg of BW	1.0 (Reference)	1.0 (Reference)	Protein intake, g/kg of BW	−4.384 (−8.841, 0.073)	−7.362 (−18.472, 3.748)
≥0.71 g/kg of BW	−0.579 (−1.275, 0.116)	0.078 (−1.475, 1.630)	≥1.00 g/kg of BW	−0.560 (−2.561, 1.441)	0.412 (−1.189, 2.012)	≥1.00 g/kg of BW	−0.116 (−1.627, 1.396)	0.624 (−1.142, 2.391)			

* Models were adjusted for age, sex, body mass index, energy intake, physical activity, active smoking, fasting state, and antidiabetic (for blood glucose) therapy. Abbreviations: BMI, body mass index, BW, body weight, CI, confidence interval.

## Data Availability

Data are available upon reasonable request from the corresponding authors.
